# Interleaved Whole Brain ^23^Na‐MRI and ^31^P‐MRSI Acquisitions at 7 Tesla

**DOI:** 10.1002/nbm.70012

**Published:** 2025-02-16

**Authors:** Zahra Shams, Jiying Dai, Mark W. J. Gosselink, Hans J. M. Hoogduin, Wybe J. M. van der Kemp, Fredy Visser, Dennis W. J. Klomp, Jannie P. Wijnen, Evita C. Wiegers

**Affiliations:** ^1^ Center for Image Sciences University Medical Center Utrecht Utrecht The Netherlands; ^2^ Tesla Dynamic Coils B.V Zaltbommel The Netherlands; ^3^ Philips Healthcare Best The Netherlands

**Keywords:** ^23^Na‐MRI, ^31^P‐MRSI, interleaved x‐nuclei scan, SAR limits, scan efficiency, ultra‐high field

## Abstract

Non‐^1^H nuclei magnetic resonance spectroscopy (MRS) offers insights into metabolism, which may aid for example early stages of disease diagnosis, tissue characterization or therapy response evaluation. Sodium MRI can provide valuable information about tissue health and cellular function. When combined with ^31^P MR spectroscopic imaging (MRSI), complementary metabolic information on energy metabolism and cell proliferation can be obtained. However, sensitivity challenges stemming from low natural abundances and low gyromagnetic ratios of different nuclei have hindered progress. Besides, due to hardware constraints, different nuclei are often studied separately, and the need for dedicated hardware for x‐nuclei imaging hampers clinical efficiency and patient‐friendly assessments. This work introduces an interleaved acquisition scheme for 3D ^31^P‐MRSI and 3D radial ^23^Na‐MR imaging (^23^Na‐MRI) at 7 Tesla (7T) and demonstrates the feasibility of interleaving these two nuclei acquisitions. The interleaved protocol effectively merged ^31^P‐MRSI with ^23^Na‐MRI, while remaining within specific absorption rate (SAR) limits. Results revealed comparable signal‐to‐noise ratios (SNRs) and spectral quality between interleaved and non‐interleaved scans, highlighting the approach's efficiency without compromising data quality.

## Graphical abstract

Sodium MRI can provide valuable information about tissue health and cellular function. When combined with ^31^P MR spectroscopic imaging (MRSI), complementary metabolic information on energy metabolism and cell proliferation can be obtained. This study introduces interleaved 3D ^31^P‐MRSI and 3D radial ^23^Na‐MR imaging at 7T, demonstrating the feasibility of interleaving these two nuclei acquisitions. Results confirm similar SNR and spectral quality of data with and without interleaving, affirming this efficient approach without compromising data integrity.

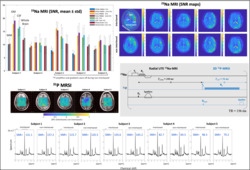

**Abbreviations**
ATP
: adenosine triphosphate
CSF
: cerebrospinal fluid
CSI
: chemical shift imaging
FA
: flip angle
FID
: free induction decay
GM
: gray matter
MRS
: magnetic resonance spectroscopy
MRSI
: MR spectroscopic imaging
PME
: phosphomonoester
PCr
: phosphocreatine
PDE
: phosphodiester
Pi
: inorganic phosphate
ppm
: part per million
ROI
: region of interest
SAR
: specific absorption rate
SNR
: signal to noise ratio
SPMU
: software power monitoring unit
STD
: standard deviation
TE
: echo time
TR
: repetition time
UTE
: ultrashort echo‐time
WM
: white matter

## 1 Introduction

X‐nuclei MRS/MRI is used to study tissue metabolism. Different x‐nuclei MRS(I) and MRI, such as ^31^P‐MRSI and ^23^Na‐MRI can provide complementary metabolic information. ^31^P‐MRSI provides insight into cell energy status, which is altered in for example tumors or neurodegenerative diseases, or it can be used to detect more subtle changes in brain energy metabolism during a visual stimulus [1, 2, 3]. Moreover, ^31^P‐MRSI provides information about intra‐ and extracellular pH as well as phospholipid metabolism enabling early therapy response assessment in cancer [4, 5, 6]. ^23^Na‐MRI can be used to estimate cell integrity and tissue viability of a healthy or diseased brain; total brain sodium content has been shown to be a biomarker of neurodegeneration in multiple sclerosis and Alzheimer's diseases [7, 8, 9], and a biomarker of viable, but hypoxic at‐risk tissue in stroke [10, 11, 12]. Furthermore, ^23^Na‐MRI has shown sensitivity to treatment‐induced effects in glioblastomas which demonstrates its predictive potential in comparison to conventional clinical data [13].

For studies focusing on pathophysiology, the combined information about all these different metabolic processes could aid in understanding of the disease [14, 15, 16, 17]. However, these different mechanisms are not very often studied in the same patient due to MR hardware and acquisition time restrictions. The independent evolution of non‐coupled x‐nuclei during an MR experiment allows for simultaneous acquisition. The ability to acquire data from multiple nuclei at the same time, may reduce the overall scanning duration. This results in protocols more acceptable for patients, and it reduces the possibility of motion artifacts during long scan times compared to separate scans of each nucleus. Besides, data collection from transient states or temporary conditions, for instance during a stimulus or treatment, is often difficult or impossible to replicate in many studies [2, 15, 16, 18, 19]. These challenges can be mitigated by acquiring data from multiple nuclei within a single scan session and in a single scan. Different techniques have been offered so far to facilitate multi‐nuclear acquisition of proton with other nuclei. These techniques focus on either a simultaneous acquisition where two resonance frequencies are received at the same time [20, 21, 22, 23], or an interleaved acquisition where signal from one nucleus is acquired while the longitudinal magnetization from the other nucleus recovers during the TR of the interleaved sequence [15, 18, 24, 25, 26, 27, 28].

Although not often used in clinical MR studies, the feasibility of interleaved acquisition of multiple nuclei has been demonstrated across various field strengths. These investigations encompassed combined acquisitions involving ^1^H (MRI and/or MRS(I)) with nuclei such as ^31^P, ^23^Na, ^19^F, ^2^H, or ^13^C both in human and animal body and brain studies [20, 21, 24, 25, 26, 29, 30]. Furthermore, interleaved acquisitions of two x‐nuclei other than ^1^H have also been explored. Notable examples include interleaved acquisitions of ^13^C and ^31^P in human muscle [31], as well as non‐human studies involving ^31^P, ^23^Na, and ^19^F MRS [32, 33, 34, 35, 36, 37, 38]. The realization of these multi‐nuclear acquisitions often required specific adjustments in software [39, 40] or the transmission/receive pathways, including the incorporation of additional or modified RF amplifiers [41], modified or supplementary spectrometers [24, 42, 43], or separate transmitters/receivers coils [16, 19]. For a comprehensive overview of these techniques, an insightful review paper by Lopez Kolkovsky et al. 2022 is available for reference [44].

To enable full metabolic characterization of the brain within one scan session, a quintuple‐tuned head coil was developed recently [45]. With this coil we can acquire data from ^1^H, ^31^P, ^23^Na, ^13^C, and ^19^F in one single setup. This enables imaging complementary metabolic processes within the same time frame. To date, there has been an absence of research dedicated to investigating the interleaved acquisition of ^23^Na‐MRI and ^31^P‐MRSI. Given the potential of combining ^23^Na‐MRI and ^31^P‐MRSI to offer a more comprehensive understanding of physiological and pathological alterations at a biological level, consequently facilitating the exploration of advanced diagnostic and therapeutic avenues, the primary objective of this study is to assess the practicality and feasibility of interleaving the acquisition of ^23^Na MRI and ^31^P MRSI. To achieve this, we interleaved acquisitions of ^31^P free‐induction decay MRSI (FID‐MRSI) with ultrashort echo‐time (UTE) ^23^Na imaging at 7T within the same overarching TR. To ascertain the absence of interactions between the two nuclear spin perturbations, we conducted both qualitative and quantitative analyses, comparing the outcomes of the interleaved scan with those of sequentially conducted scans of equivalent duration.

## 2 Methods

MR experiments were performed on a 7 Tesla MR scanner (Achieva; Philips, Best, Netherlands) equipped with a two‐channel ^31^P birdcage coil integrated in the bore used as RF transmitter and a quintuple tuned RF coil with 8 transmit/receive ^1^H/^19^F dipole antennas, combined with a fifteen‐loop receive‐only array double tuned to ^31^P and ^23^Na frequencies and switchable to ^13^C (120.7, 78.8 and 75 MHz, respectively). A Helmholtz clamp [46] was used for ^23^Na excitation.

**FIGURE 1:**
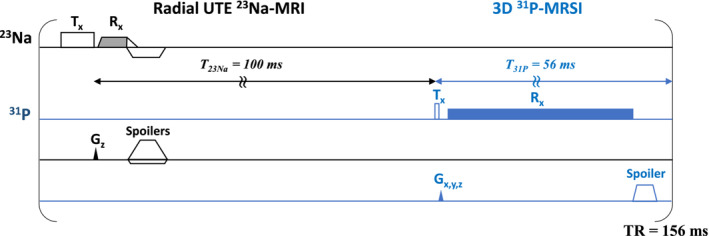
(A) Pulse sequence diagram for the interleaved acquisition of ^23^Na MRI (TE = 0.19 ms) and ^31^P MRSI (TE = 0.67 ms). Signal from phosphorus is acquired while the longitudinal magnetization from sodium recovers during the TR of the interleaved sequence (156 ms). Because of radial sampling, an echo time shorter than the sodium fast T_2_ component (T_2_ ranging from 0.5 to 5 ms [47]) was achieved.

^31^P‐MRSI acquisition was interleaved with a ^23^Na sequence using a 3D radial acquisition, as depicted in Figure 1. The software implementation was previously described by [39, 40]. Based on global specific absorption rate (SAR) limits for the head (3.2watt/kg) and using an error margin of 10%, the maximum allowed average forward power for both transmit coils, $P_{\text{aveforward},\max\left(31P\right)}$ and $P_{\text{aveforward},\max\left(23\mathrm{Na}\right)}$, is determined (15 watt (W) for the ^23^Na local transmit coil and 120 W for the ^31^P bore coil). The transmitted power for each transmit coil is monitored as the ratio of average forward power ($P_{\text{aveforward}\left(31P\right)}$ and $P_{\text{aveforward}\left(23\mathrm{Na}\right)}$) over its maximum allowed average forward power times 100%. This ratio, summed for both transmit coils (the summed power ratio) should never exceed 100% (Equation 1).

(1) $$\left(\frac{P_{\text{aveforward}\left(23\mathrm{Na}\right)}}{P_{\text{aveforward},\max\left(23\mathrm{Na}\right)}}+\frac{P_{\text{aveforward}\left(31P\right)}}{P_{\text{aveforward},\max\left(31P\right)}}\;\right)\leq 100\%$$

The RF pulses of both transmit chains (^23^Na and ^31^P) were monitored by using the directional couplers in the amplifiers. The sampled waveforms are converted to average forward power by the software power monitoring unit (SPMU) of the MR scanner. The reflected power was not taken into account. The optimization process begins with the most SAR‐demanding part of the sequence, ensuring the maximum SNR per unit of time is achieved. The ^23^Na‐MRI scan is more SAR demanding than the ^31^P‐MRSI scan attributable to the short repetition time (TR) required by ^23^Na considerably shorter T_1_ relaxation time. Consequently, a larger proportion of the summed ratio in Equation 1 should typically be allocated to the ^23^Na‐MRI scan. When using the most SAR‐efficient RF pulse, such as a block pulse with no negative side lobes, the only way to reduce SAR while maintaining optimization for SNR per unit time is to stretch the pulse. However, ^23^Na also exhibits very short T_2_ relaxation time, so optimization must balance a reduction in SNR per unit time by either lengthening the TR or stretching the RF pulse. Given the available RF power of the setup, we observed that the shortest possible RF pulse achievable for ^23^Na resulted in a relatively long TE. Consequently, this RF pulse was fixed. Alternatively, a substantially shorter TR could have been selected, reducing the (Ernst) flip angle. However, within the realistic range of scan parameters that allows sufficient time for data acquisition, this would only lead to an overall increase in SAR. The remaining SAR, caused by the optimal yet very low flip angle of ^31^P, is primarily a consequence of fixing the RF pulse for ^23^Na. Further optimization of the sequence parameters was not considered, as this could introduce complications due to variances in T_1_ and T_2_, each of which would necessitate slightly different optimized TR and flip angles. A more detailed analysis of sequence optimization and the effects of TR and pulse duration selection is provided in the Appendix. Given the available ^23^Na RF peak power of 571 W at the coil port, which provides a maximum B_1_^+^ of 6 μT, and assuming Ernst angle excitation, the shortest achievable RF pulse duration was 3.3 ms. With this limitation on B_1_^+^ and the consequent adjustment of the pulse duration, the highest SNR per unit time was achieved at a TR of 156 ms.

To accommodate some SAR for ^31^P, we lowered the flip angle for ^23^Na from the Ernst angle of 87.5° to 80°. This adjustment freed up 20% of the SAR at the cost of approximately 2% reduction in ^23^Na SNR. Therefore, the time averaged power of the whole sequence, governing the SAR, never surpassed the summed power ratio limit for the volume transmitter for ^31^P and the ^23^Na Helmholtz clamp transmitter. After optimizing the TR and pulse duration parameters for ^23^Na‐MRI, the Ernst angle for ^31^P‐MRSI sequence was determined using the obtained TR of 156 ms. Sequence design parameters can be seen in Table 1.

**TABLE 1:** Interleaved ^23^Na‐^31^P sequence design parameters. $P_{\text{aveforward},\max}$, maximum allowed average forward power; $P_{\text{peak}}$, peak power; $P_{\text{aveforward}}$, average forward power; B_1_^+^, amplitude of B_1_ transmit; FA, flip angle; $\tau_{p}$, RF pulse duration; TR, repetition time; NSA, number of signal averages.

	$P_{\text{aveforward},\max}$ (W)	$P_{\text{peak}}$ (W)	$\frac{P_{\text{aveforward}}}{P_{\text{aveforward},\max}}$ (%)	B_1_^+^ (μT)	FA (°)	$\tau_{p}$ (ms)	TR (ms)	NSA
^**23**^ **Na**	15	571	80	6	80	3.3	156	1
^**31**^ **P**	120	4050	20	6	16	0.43	156	40

*T*_*31P*_ and *T*_*23Na*_ (see Figure 1) which are the time interval between the phosphorus excitation and the end of TR, and the time interval between sodium and phosphorus excitations were subsequently utilized in the preparation of protocols for each of the nuclei. Considering the shortest possible TR for a safe level of average power to be 156 ms, *T*_*23Na*_ was empirically set to 100 ms, leading to the subsequent setting of *T*_*31P*_ to 56 ms.

### 2.1 Acquisitions

Our experiments involving human subjects were performed according to the MRI protocol development protocol which was approved by the institutional ethical review board (NedMec NL53099.041.15). We performed in vivo measurement on five healthy volunteers (2 females, 3 males, 33 ± 8 years) after providing written informed consent.

^23^Na‐MRI was acquired with an ultrashort echo‐time (UTE) imaging sequence using 3D radial k‐space sampling of the FID with the following scan parameters: TE = 0.19 ms, TR = 156 ms, FA = 80°, FOV = 256 × 256 × 160 mm^3^, in‐plane resolution = 4 × 4 mm^2^, slice thickness = 10 mm, number of projections = 5900, radial percentage = 230% and readout time per spoke = 7.9 ms.

^31^P‐MRSI was acquired with a 3D FID‐CSI sequence: matrix size = 11 × 11 × 9, voxel size of 2 × 2 × 2 cm^3^, TR = 156 ms, FA (Ernst angle) = 16°, Hamming‐weighted k‐space sampling with 40 number of averages, 256 data points, spectral width = 5000 Hz. The number of averages of the MRSI sequence was optimized to ensure that both ^31^P MRSI and ^23^Na MRI scans were completed after an equal number of TR repetitions.

Total acquisition time of the interleaved ^23^Na‐^31^P acquisition was 15 min (min) and 19 s (s).

For comparison, reference data from each individual ^23^Na/^31^P nucleus were acquired in the same scan session. The first two reference datasets were obtained by repeating the same interleaved scans twice without changing the scan parameters, but with the transmit amplifiers for the other nucleus switched off. For the last three volunteers, ^31^P and ^23^Na scans were acquired separately to avoid the potential impact of additional active gradients that would be used for acquiring the other nucleus.

The acquisition parameters for the other scans are as follows:

^1^H‐MRI anatomical images were acquired using T1 3D turbo fast spin echo (TFE) sequence with the following parameters: FOV = 200 × 250 × 180 mm^3^, voxel size = 1 × 1.35 × 2 mm^3^, matrix size = 200 × 186 × 180, slice orientation = sagittal, sense factor = 2, TFE factor = 250, FA = 6°, TR = 8 ms, TE = 1.97 ms, NSA = 2, acquisition time = 3 min 38 s.

For B_0_ shimming, we acquired a 3D B_0_ map (3D gradient echo) with these sequence parameters: FOV = 250 × 203 × 150 mm^3^, voxel size = 2.34 × 2.34 × 6 mm^3^, FA = 5°, TR = 10 ms, TE = 1.74 ms, ∆TE = 1 ms, acquisition time = 43 s. Second‐order shim settings were optimized over the whole brain using the MR Code software (TeslaDC, Zaltbommel, The Netherlands). All the protocol parameters are listed in Appendix Table A.1.

### 2.2 Data Processing

Sodium radial data were reconstructed and consequently channel combined using scanner software. Reconstruction and processing steps of raw 3D ^31^P MRSI data were as follows: k‐space apodization with hamming filter, averaging, spatial fast Fourier transform (FFT), zero‐ and first‐order phasing, PCA‐based denoising [48] and Roemer channel combination [49]. OXSA Matlab code v1.0 was used for analysis of the 3D ^31^P data [50] and calculating the amplitude and linewidth of phosphocreatine (PCr) signal.

### 2.3 Data Analysis

**FIGURE 2:**
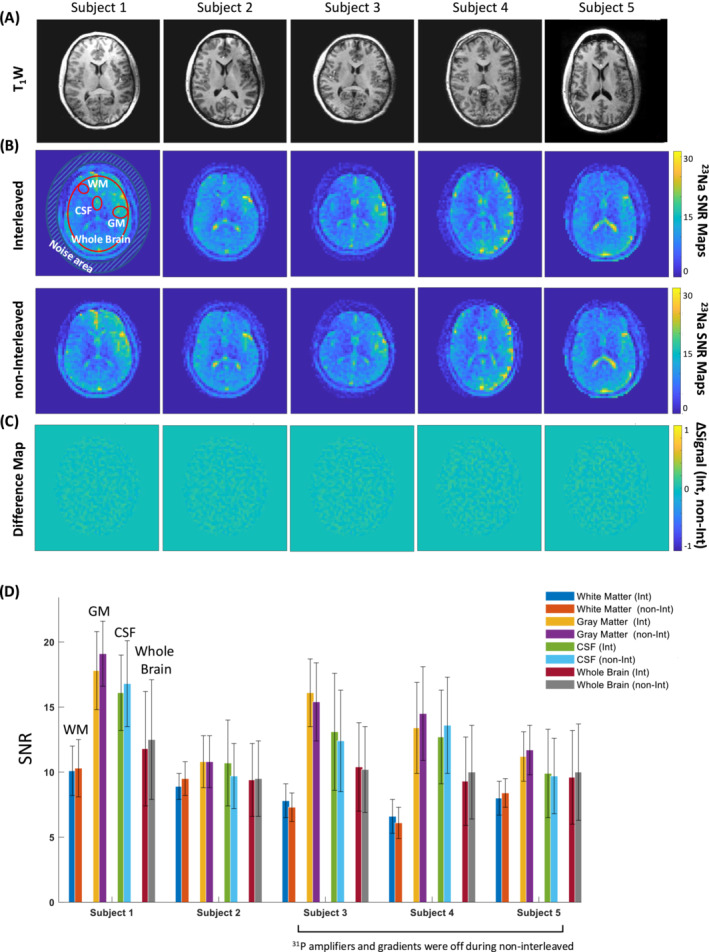
Comparison of interleaved and non‐interleaved sodium scans collected from five subjects whose anatomical scans are shown in (A). (B) SNR maps of UTE sodium MRI data acquired using interleaved (top row) and non‐interleaved (bottom row) strategies. (C) The normalized difference maps of sodium MRI for all subjects. Each of the sodium MRI images were normalized to the maximum intensity of the image. (D) The bar plots illustrate the mean ± STD of ^23^Na SNR within four distinct regions of interest (ROIs): white matter (WM), gray matter (GM), cerebrospinal fluid (CSF), and a broader brain ROI (accompanied by the corresponding anatomical image) for both interleaved and non‐interleaved sodium images. Non‐interleaved sodium scans for Subjects 3, 4 and 5 were acquired while ^31^P‐related amplifiers and gradients were switched off. Int, interleaved; non‐int, non‐interleaved.

To evaluate the signal‐to‐noise ratio (SNR) of the ^23^Na‐MRI, we defined three regions of interest (ROI). These regions predominantly encompassed cerebrospinal fluid (CSF), gray matter (GM), and white matter (WM). Additionally, a larger ROI covering the majority of inner‐brain structures was also established. The noise value was determined by calculating the standard deviation of the area situated outside the head, as depicted in Figure 2. UTE ^23^Na‐MRI SNR maps were created by normalizing the ^23^Na‐MRI images based on the background noise's standard deviation. Difference maps for sodium MRI were generated by subtracting the images normalized to their maximum values.

SNR of ^31^P‐MRSI data was calculated as the ratio of the amplitude of the PCr signal to the standard deviation of a signal free noise region (15 to 20 ppm). The amplitude and linewidth of PCr signal were calculated. The resulting PCr amplitude maps for each interleaved and non‐interleaved ^31^P‐MRSI scans were normalized to the maximum PCr amplitude. These normalized maps were then subtracted from each other to create the PCr difference maps.

## 3 Results

**FIGURE 3:**
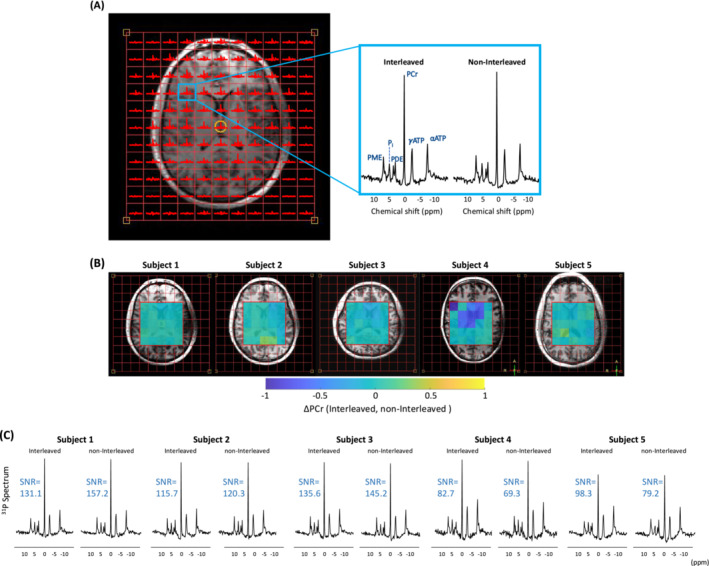
Comparison of ^31^P data acquired from interleaved and non‐interleaved. (A) ^31^P spectra from a voxel within the 3D CSI grid obtained via interleaved and non‐Interleaved acquisitions. Spectra were normalized to the same noise level. (B) Difference maps of PCr signal normalized to their maximum values between the interleaved and non‐interleaved scans for all five subjects. (C) Examples of ^31^P spectra and their corresponding SNR values before and after interleaving. The SNR is calculated after PCA‐based denoising and is based on the PCr amplitude.

Figure 2 presents T_1_‐weighted images alongside sodium SNR maps from both interleaved and non‐interleaved ^23^Na‐^31^P scans, all corresponding to the central slice of the T_1_‐weighted image for five subjects. The two types of scans show similar sodium SNR maps, with minimal differences observed between the interleaved and non‐interleaved maps (Figure 2B,C). Quantitatively, the mean and standard deviation (STD) of the SNR for the regions of interest (ROIs) in white matter (WM), gray matter (GM), cerebrospinal fluid (CSF), and the whole brain were comparable for both interleaved and non‐interleaved scans and for all five subjects (Figure 2D). Figure 3 depicts the ^31^P spectra of a sample voxel extracted from the central slice of the 3D ^31^P‐MRSI matrix, showing similar spectral quality achieved through interleaved and non‐interleaved scans (Figure 3A). Figure 3B presents a quantitative analysis of interleaved and non‐interleaved ^31^P scans, demonstrating that all subjects, but Subject 4, have similar PCr maps between the two scanning methods. Subject 4 displayed notable differences across multiple voxels. Example spectra from interleaved and non‐interleaved scans for each subject, along with corresponding SNR levels, show comparable values between the two scan types (Figure 3C).

Table 2 summarizes the PCr line width and SNR (based on PCr signal) of the voxels within the ROI (green square) across all volunteers for the center slice of the CSI matrix. Mean linewidth and mean SNR of the PCr signal within the ROI were similar between the interleaved and non‐interleaved scans.

**TABLE 2:** Mean (±STD) PCr linewidth and mean (±STD) SNR within the delineated region of interest (ROI) in Figure 3B for all five subjects. Signal amplitude and line width measurements (in Hz) were obtained through fitting analysis using OXSA‐AMARES.

(A) Linewidth of PCr signal in the ROI		
Subject	Interleaved mean ± std (Hz)	Non‐interleaved mean ± std (Hz)
**#1**	13.1 ± 1.2	13.2 ± 1.2
**#2**	14.5 ± 1.7	15.0 ± 2.0
**#3**	15.9 ± 1.0	14.5 ± 1.3
**#4**	16.1 ± 2.5	12.2 ± 2.6
**#5**	20.3 ± 5.7	22.4 ± 7.1

(B) SNR based on PCr signal in the ROI		
Subject	Interleaved mean (rms) ± std	Non‐interleaved mean (rms) ± std
**#1**	29.7 ± 3.8	31.5 ± 3.5
**#2**	24.0 ± 5.2	23.0 ± 4.2
**#3**	29.3 ± 8.1	25.7 ± 3.7
**#4**	32.9 ± 6.2	27.4 ± 2.6
**#5**	20.7 ± 2.3	20.4 ± 4.4

## 4 Discussion

In this study, we introduced an interleaved ^31^P‐MRSI and ^23^Na‐MRI acquisition within a single scan. The objective was to investigate the feasibility of interleaving the ^31^P‐MRSI sequence, which exhibits a low SAR demand, with the SAR‐demanding ^23^Na MRI. The results of our comparative analysis between the interleaved and the non‐interleaved scans (i.e., with the same scan protocol) revealed that the interleaved approach per se did not lead to reduction in SNR for either nucleus or reduced ^31^P spectral quality, while time‐efficiency improved. These findings demonstrate the benefits of an interleaved acquisition strategy, allowing for efficient multi‐nuclear imaging without compromising the data quality of individual nuclei.

Both ^31^P‐MRSI and ^23^Na‐MRI hold significance in clinical settings due to its capability to provide insights into tissue health, cellular viability, and metabolic changes. However, clinical adoption not only has been hindered by the challenges posed by the low natural abundance and low gyromagnetic ratio of different nuclei causing a low sensitivity, but also by the need for non‐standard hardware and time‐consuming imaging protocols. Ultra‐high field strengths have been increasingly explored for x‐nuclei MRI which offer numerous benefits, including increased SNR and enhanced spectral resolution. These advantages lead to improved image quality, higher sensitivity to metabolic changes in tissue, and better biophysical insights. Additionally, advancements in ultra‐high field MRI technology, exemplified by innovative hardware like radiofrequency coils [45] and software frameworks [39, 40], enable concurrent imaging and spectroscopy. However, to allow seamless integration of various nuclei in standard MR imaging there is a need for a multi‐nuclear assessment within a patient‐friendly time frame. This study primarily aimed to achieve efficient and simultaneous acquisition of ^23^Na MRI and ^31^P MRSI data.

In the presented interleaved scheme, we employed a relatively low B_1_ (compared to the short T_2_* of ^23^Na) in a rectangular‐shaped pulse—one of the most SAR‐efficient RF pulses available. Any further reduction in SAR would compromise the echo time, thereby reducing the SNR. Recognizing the importance of SAR as a critical parameter, the reasoning outlined here supports and justifies our choice of RF pulses in this study. However, using a very long RF pulse duration may result in strong B_0_ artifacts. In this study, we employed a peak power of 571 W to achieve a B_1_ of 6 μT with a 3.3 ms pulse for ^23^Na excitation. The bandwidth of the 3.3 ms pulse (~300 Hz) is still adequate to excite the spins in the well B_0_‐shimmed brain (~40 Hz).

Regarding the efficiency of the proposed interleaved scan, the gain in scan time is not a factor of two, as we could potentially opt for a shorter TR for ^31^P, of around 60 ms. The effect of the longer TR used in this study is balanced by using a larger FA (Ernst angle) to maintain a similar SNR per unit of time. Given the optimal TRs for the non‐interleaved ^23^Na and ^31^P protocols are 160 ms [13] and 100 ms, respectively, as well as the optimal number of averages of ^31^P protocol to be 28 instead of 40 used in our interleaved sequence, a 25% increase in scan efficiency was achieved by the interleaved scan protocol.

The interleaved imaging protocol introduced in this study effectively integrated ^31^P‐MRSI with a 3D radial ^23^Na sequence. The selection of the TR for the interleaved sequence was critical to stay well within SAR limits. We carefully determined the minimum TR, ensuring that the time‐averaged power of the entire sequence never exceeded the combined limits of the average power for both the ^31^P volume transmitter and the ^23^Na transmitter, thereby ensuring the safety and reliability of the interleaved sequence. To optimize scan efficiency while upholding safety standards, future improvements could involve the incorporation of interleaved SAR estimation tailored for actual scanning conditions. This would dynamically adjust RF power limits based on the specific combination of scans being interleaved. Thus, SAR management for each sequence could potentially be optimized automatically. This, in turn, could lead to a reduction in scan time and thus improve patient comfort. In our study, combining the two scans with different SAR contributions was straightforward due to the considerably higher SAR demand of the ^23^Na scan compared to the ^31^P scan. Once optimized for the ^23^Na scan, the ^31^P scan had minimal impact on the already optimized RF power setting.

We observed significant differences in PCr signal between interleaved and non‐interleaved scans in certain voxels (Figure 3B). However, these differences were not consistent across all volunteers. For example, Subjects 1 and 3 exhibited minimal differences in PCr signal between the two scan types, while Subject 4 showed a notable variation across several voxels. Given this inconsistency, we do not attribute the variability in PCr signal between interleaved and non‐interleaved scans to the interleaving procedure itself. Instead, this difference may be due to interscan subject movements.

It was expected that cerebrospinal fluid (CSF) would appear much brighter in the ^23^Na images in Figure 2 and have a significantly higher SNR compared to GM. As the receive coils for both nuclei are surface coils, we expect a receive sensitivity pattern with high signal at the edge and lower signal in the middle. Therefore, this pattern should be the matter of receive B_1_.

The primary aim of this study was to compare ^31^P‐MRSI and ^23^Na‐MRI data obtained with an interleaved scan to non‐interleaved scans. The comprehensive quantification of the data was therefore beyond the scope of this study, and processing steps such as B_0_ and B_1_ inhomogeneity correction, were not performed. Given that these inhomogeneities persists consistently between non‐interleaved and interleaved scan outcomes, it does not alter the conclusions drawn from the results presented here.

The proposed approach of our study has the potential to extend to other nuclei, including various combinations with ^13^C, ^19^F, and ^1^H. Leveraging the quintuple‐tuned RF coil, the interleaved scheme offers the opportunity to incorporate more than two nuclei, which can substantially reduce overall scanning time in clinical settings.

## 5 Conclusion

In this study we have shown that, by interleaving a ^23^Na‐MRI with ^31^P‐MRSI and executing them effectively in a single scan, it is possible to obtain sodium images and phosphorus MRSI of the brain within 15 min. By carefully adjusting the repetition time and employing appropriate flip angles for a safe level of total average power, we achieved effective interleaving of the sodium and phosphorus sequences. The results highlighted the potential for efficient and simultaneous multi‐nuclear imaging without compromising the data quality of MR images of individual nuclei.

## Conflicts of Interest

Jiying Dai declares a financial interest related to the subject matter of this paper through employment with Tesla Dynamic Coils B.V., the Netherlands. F. Visser holds a partial employment position with a commercial organization, Philips Healthcare, Best, the Netherlands. The remaining authors declare no conflicts of interest.

## Figures and Tables

**FIGURE A1:**
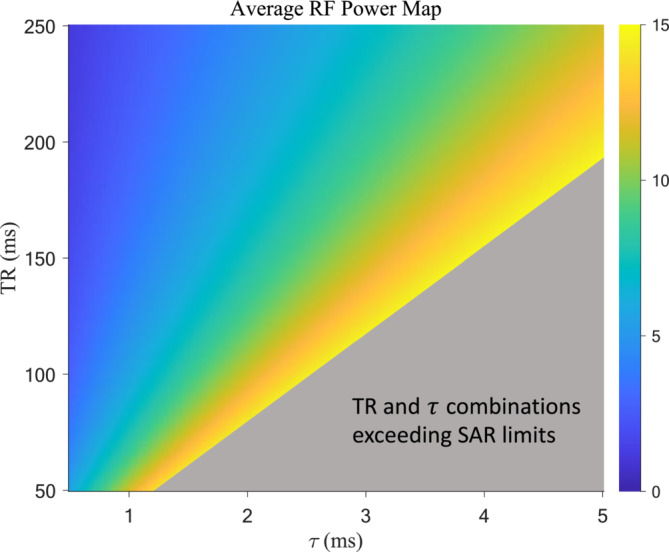
The average RF power heat map as a function of TR and pulse duration (τ), representing the allowed settings that comply with SAR limit. The gray area shows the combinations of TR and 𝜏 that exceed the SAR thresholds. The colorbar unit is in watt.

**FIGURE A2:**
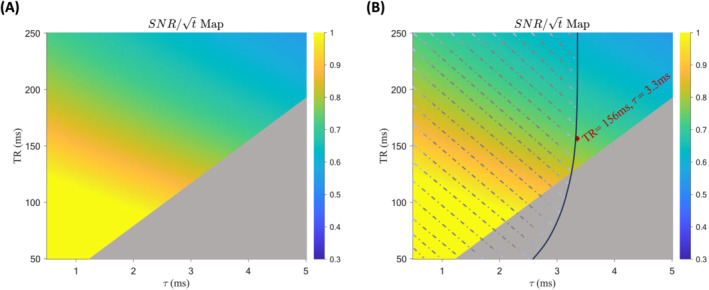
SNR per unit time as a function of different TR and pulse duration (τ) values is shown in (A) after applying SAR thresholding, and in (B) after accounting for B1 constraints. The gray area represents TR and τ combinations that exceed the SAR thresholds, while the dashed area indicates invalid combinations due to B1 constraints. The red marker in (B) highlights the selected TR and pulse duration used in our experiments.

## Data Availability

The data that support the findings of this study are available from the corresponding author upon reasonable request.

## Appendix A

The sequence parameters have been optimized to maximize SNR by adjusting the TR and pulse duration (*τ*), assuming Ernst angle excitation within the constraints of the permitted SAR.

At a certain flip angle (*FA*), the RF peak power (*P*_*peak*_) is linearly related to the pulse duration: $P_{\text{peak}}\propto \frac{\mathrm{FA}}{\gamma \tau }.$ The average power (*P*_*avg*_), which determines SAR and thus limited (*P*_*ave,max*_), can be written as:

$$P_{\mathrm{ave}}=P_{\text{peak}}\times \frac{\tau_{p}}{\mathrm{TR}}<P_{\mathrm{ave},\max},$$

As stated in the Methods section, the maximum allowed average RF power for the sodium transmit coil, $P_{\mathrm{ave},\max\left(23\mathrm{Na}\right)}$, is determined as 15 W. Given the available RF peak power of 571 W at the coil port that provides a maximum B_1_ of 6 μT, and with the assumption of Ernst angle excitation, we plotted the average RF power map as a function of TR and the pulse duration (τ) (Figure A1).

We plotted the relative SNR as a function of TR and τ using Equation A1.1 and Equation A1.2, using the allowed combinations of TR and pulse duration τ (Figure A2A) to find out the optimum TR and τ. Additionally, considering the B_1_ limit, there will be a region of invalid pulse duration values caused by constraints in B_1_^+^ represented by the dashed area in Figure A2B.

(A1.1)
$$\begin{array}{c} \begin{array}{c} \mathrm{SNR}\left(\mathrm{TR},\tau \right)=\frac{1}{\sqrt{t}}\sin\left(\mathrm{FA}\right)\cdot \left(\frac{1-\exp\left(-\mathrm{TR}/T_{1}\right)}{1-\cos\left(\mathrm{FA}\right).\exp\left(-\mathrm{TR}/T_{1}\right)}\right)\cdot \left(f_{\text{short}}\exp\left(-\mathrm{TE}/T_{2,\text{short}}\right)+\left(1-f_{\text{short}}\right)\exp\left(-\mathrm{TE}/T_{2,\text{long}}\right)\right)\; \end{array} \end{array}$$

(A1.2)
$$\mathrm{FA}=\gamma B_{1}\tau$$

(A1.3)
$$B_{1}=6\mathrm{\mu T}\times {\left(\frac{P_{\text{peak}}}{571\;W}\right)}^{1/2}$$

Where $\mathrm{SNR}\left(\mathrm{TR},\tau \right)$ is *SNR* per unit time as a function of *TR* and pulse duration, $f_{\text{short}}$ is the overall contribution of short T_2_ component to the sodium signal and equal to 0.6 according to reference [47] (Madelin et al., 2014), *T*_1_ = 50 ms, *T*_2,*short*_/*T*_2,*long*_ = 5 ms/20 ms, sodium gyromagnetic ratio $\gamma =70.761\times 10^{6}\mathrm{rad}s^{-1}T^{-1}$.

The *TR* and pulse duration were chosen as depicted in Figure A2B.

**TABLE A.1:** MRSinMRS checklist. Additional columns are provided for multi‐site or multi‐sequence studies if necessary.

Site (name or number): University Medical Center Utrecht, the Netherlands		
1. Hardware	^31^P	^23^Na
a. Field strength [T]	7 T	7T
b. Manufacturer	Philips	Philips
c. Model (software version if available)	Achieva	Achieva
d. RF coils: nuclei (transmit/receive), number of channels, type, body part	Transmit: two‐channel ^31^P birdcage coil integrated in the bore of the MR system Receive: 15‐loop receive‐only array double tuned to ^31^P and ^23^Na frequencies	Transmit: ^23^Na Helmholtz clamp Receive: 15‐loop receive‐only array double tuned to ^31^P and ^23^Na frequencies
e. Additional hardware	−	−
2. Acquisition		
a. Pulse sequence	FID‐MRSI	3D radial acquisition
b. Volume of interest (VOI) locations	Whole brain	Whole brain
c. Nominal VOI size [cm^3^, mm^3^]	20 x 20 x 20 mm^3^	4 x 4 x 10 mm^3^
d. Repetition Time (TR), Echo Time (TE) [ms, s]	TR 156 ms TE 0.62 ms	TR 156 ms TE 0.19 ms
e. Total number of Excitations or acquisitions per spectrum	40 NSA	1 NSA
f. Additional sequence parameters (spectral width in Hz, number of spectral points, frequency offsets) If STEAM: Mixing time (TM) If MRSI: 2D or 3D, FOV in all directions, matrix size, acceleration factors, sampling method	Spectral width: 5000 Hz Number of spectral points: 256 Frequency offset: 0 ppm FOV: 220 x 220 x 180 mm^3^ Matrix size: 11 x 11 x 9 Acceleration: hamming weighted k‐space sampling	FOV: 256 x 256 x 160 mm^3^ Number of projections 5900 Radial percentage 230% Readout time per spoke 7.9 ms UTE Trajectory delay 20 μs
g. Water suppression method	−	−
h. Shimming method, reference peak, and thresholds for “acceptance of shim” chosen	Whole brain 2nd order B_0_ shimming Reference peak: H_2_O	Whole brain 2nd order B_0_ shimming Reference peak: H_2_O
i. Triggering or motion correction method (respiratory, peripheral, cardiac triggering, incl. device used and delays)	−	−
3. Data analysis methods and outputs		
a. Analysis software	AMARES (OXSA Matlab toolbox)	Matlab
b. Processing steps deviating from quoted reference or product	k‐space apodizationzero‐ and first order phase correction PCA‐based denoising	−
c. Output measure (e.g., absolute concentration, institutional units, ratio)processing steps deviating from quoted reference or product	Amplitude (a.u.) and linewidth (Hz) of phosphocreatine (PCr)	SNR of ^23^Na‐MRI
d. Quantification references and assumptions, fitting model assumptions	Lorentzian lineshapes	—
4. Data quality		
Reported variables (SNR, linewidth (with reference peaks))	Linewidth PCr between 13.1–22.4 Hz SNR PCr between 20.4–32.9	SNR ^23^Na between 10.6–20.4
b. Data exclusion criteria	*−*	*−*
c. Quality measures of postprocessing model fitting (e.g., CRLB, goodness of fit, SD of residual)	*−*	*−*
d. Sample spectrum	Figure 3	Figure 2
